# Influence of Core Configuration on the Flexural Behavior of Lightweight CFRP Sandwich Panels in Drone Design

**DOI:** 10.3390/polym18141682

**Published:** 2026-07-08

**Authors:** Mihai Parparita, Paul Bere, Razvan Udroiu, Mircea Cristian Dudescu

**Affiliations:** 1 Manufacturing Engineering Department, Technical University from Cluj-Napoca, 400641 Cluj-Napoca, Romania; mihai.parparita@campus.utcluj.ro; 2 Manufacturing Engineering Department, Transilvania University of Brasov, 500036 Brașov, Romania; 3 Mechanical Engineering Department, Technical University from Cluj-Napoca, 400641 Cluj-Napoca, Romania; mircea.dudescu@rezi.utcluj.ro

**Keywords:** sandwich, CFRP, lightweight core, bending, microscopy, drone structure

## Abstract

Sandwich structures have gained much interest in drone manufacturing structures based on their lightweight design and excellent mechanical characteristics. In this work, a new solution for lightweight drone wing structures consisting of a thin sandwich skin, a main spar, and ribs was proposed. Seven sandwich structures based on prepreg-based CFRP skins and different cores were proposed for the wing drone sandwich skin. Thus, sandwiches with different chemical configurations and densities, such as ROHACELL 51, AIREX T92.100, balsa, AIREX R82.150, AIREX C71.75, NOMEX ECA-I, and Soric XF, were autoclave-manufactured and investigated. All the samples were tested under three-point bending. Also, microscopic analysis of the fracture zones was performed to establish a direct link between macroscopic flexural behavior and local failure mechanisms. A statistical analysis based on ANOVA with Box–Cox transformation followed by Tukey’s Honestly Significant Difference test was performed for flexural strength and flexural modulus. The results show that the sandwiches containing Soric XF foam with 62.5 kg/m^3^ density had the best mechanical properties, with a 71.66 MPa flexural strength and a 10,039 MPa flexural modulus.

## 1. Introduction

Sandwich structures are widely adopted in lightweight structural applications for aerospace, automotive, and marine components [1,2]. They are widely used in aerospace applications [3,4] due to their high stiffness-to-weight ratio, making them ideal for components such as fuselages and wings for aircrafts [5], unmanned aerial vehicles (UAVs) with fixed wings [6], gliders [7], UAVs with rotary wings [8,9], and satellite components. In this context, composite sandwich structures based on CFRP skins and lightweight cores represent an effective solution for drone airframes, offering high stiffness-to-weight ratios, damage tolerance, and design flexibility [5]. Furthermore, the potential of these structures for developing lightweight and optimized design structures is confirmed by new manufacturing methods such as additive manufacturing [10,11,12].

Sandwich structures’ design, mechanical testing, manufacturing, and maintenance can be systematically addressed through Product Lifecycle Management (PLM) methodologies across all stages of the product lifecycle [13]. The mechanical behavior of composite sandwich structures under flexural loading has been the subject of extensive experimental and numerical research. Previous studies have demonstrated that core material, density, and architecture strongly influence bending stiffness, strength, and energy absorption capacity [14,15,16].

The core configuration plays a critical role in determining the overall stiffness, strength, and failure behavior of sandwich structures [2,3]. Investigations on foam, honeycomb, and architected polymer cores have reported distinct failure modes, including core shear cracking, face-sheet fracture, local indentation, and interfacial debonding [17,18,19]. Within the literature, several works have addressed the flexural response of polymer-based sandwich panels, emphasizing the role of core properties in tailoring global mechanical performance [20]. The study by Daniel and Abot [21] showed that the mechanical response of sandwich beams is strongly influenced by the face-sheet/core interaction, with failure modes including core shear, face wrinkling, and debonding under flexural loading. Additionally, Ref. [22] demonstrated that honeycomb sandwich panels provide excellent stiffness-to-weight performance, where core geometry and material selection significantly affect bending behavior, load distribution, and overall structural stability. These characteristics highlight the importance of optimizing both skin and core properties to achieve superior mechanical performance in sandwich composite structures.

Recent studies have highlighted the significant influence of core material properties and environmental conditions on the flexural and impact performance of composite sandwich structures. Kaboglu et al. [23] demonstrated that increasing foam-core density enhances the quasi-static flexural strength and ballistic resistance of fiber-composite sandwich panels. Focusing on durability, Wu et al. [24] investigated the effects of moisture on balsa-wood-core sandwich composites under four-point bending and found that water absorption led to reduced mechanical performance and modified failure mechanisms. Liu et al. [20] combined experimental testing with numerical simulations to examine the flexural behavior of curved sandwich beams with PET foam cores, showing that structural curvature and core properties significantly affect stiffness, load-carrying capacity, and failure modes. Extending the comparison of core materials, Pach et al. [25] evaluated sandwich composites incorporating balsa, Rohacell^®^, and Nomex^®^ cores for aerospace applications, concluding that each core material presents distinct trade-offs between weight, stiffness, strength, thermal stability, and damage tolerance.

Moran et al. have investigated subcritical damage in aluminum honeycomb and CFRP sandwiches, and developed tools that can predict damage and stiffness reduction, optimizing sandwich panel designs [26]. Yang et al. demonstrated that hybrid composite pyramidal truss sandwich panels can achieve significantly enhanced damping and stiffness efficiency, making them attractive for vibration-sensitive structures [27]. Investigating foam-core configurations, Liu et al. identified the dominant flexural failure mechanisms and highlighted the critical role of core properties in determining bending performance [16]. The influence of face-sheet geometry was further examined by Naresh et al., who reported that increasing carbon/epoxy face-sheet thickness substantially improves the flexural strength and load-carrying capacity of Nomex honeycomb sandwich panels [28]. Environmental effects were addressed by Aowad et al., who showed that Arctic temperature conditions alter both the microstructural characteristics and flexural response of foam-core sandwich materials, emphasizing the need for temperature-specific design considerations [29]. Additionally, the work of da Silva and Kyriakides provided valuable insights into the compressive behavior and failure mechanisms of balsa wood, a natural core material frequently employed in lightweight sandwich constructions [30]. Collectively, these studies demonstrate that the mechanical performance of sandwich panels is strongly influenced by core architecture, face-sheet thickness, material selection, and environmental conditions, all of which must be carefully optimized to achieve superior structural efficiency.

The majority of existing studies primarily focus on macroscopic indicators such as load–displacement curves and ultimate flexural strength, and the connection between core characteristics, observed flexural behavior, and fracture mechanisms at the microstructural level remains insufficiently explored, particularly for CFRP sandwich structures incorporating different polymeric cores. Also, most of the research is limited to a small number of core configurations or focuses on a single core material system. Consequently, additional research is needed to establish structure–property relationships that support the optimization of lightweight sandwich composites for practical engineering applications, including UAVs and drone structures.

The main contribution of this paper is in the field of lightweight structural material selection, namely finding a new solution for drone wing structure configuration. The proposed configuration of the wing structure consists of a sandwich skin, a main spar, and ribs. The main advantage of this wing structure is the reduction in the number of ribs compared to the traditional wing configuration. In this way, the proposed structure will be lighter than the traditional wing structure. The purpose of this paper is to investigate thin sandwich skins used for UAV wings and find an optimal solution. Thus, CFRP sandwich structures with different core materials such as PMI, PET, PEI, PVC, polyester, wood and Nomex were taken into consideration, and mechanically tested at bending. The novelty of the present work has been explicitly highlighted as the systematic experimental comparison of seven different lightweight core types integrated into CFRP sandwich panels and evaluated through identical three-point bending tests. This approach provides a comprehensive assessment of the influence of core configuration on the flexural behavior of sandwich structures intended for UAV applications. Three-point bending tests are combined with post-fracture microscopic examination of the skins, cores, and interfacial regions to establish a direct link between global flexural response and local damage evolution.

## 2. Materials and Methods

### 2.1. Materials

Sandwich specimens were manufactured using prepreg-based CFRP face sheets, with two layers per skin, combined with different core materials. The manufacturing process, specimen geometry, and flexural testing procedures are detailed to ensure reproducibility and to enable a consistent comparison of the flexural response and failure behavior associated with each core configuration.

Each sandwich skin was composed of two prepreg plies, stacked in a [0/90/±45] orientation, ensuring a balanced and quasi-isotropic in-plane response. This symmetric layup was selected to provide consistent mechanical behavior under flexural loading and to minimize coupling effects. The skins of all sandwich specimens were manufactured using GG245TSE-DT121H-42 prepreg based on twill-woven CFRP with a weight of 245 g/m^2^ and an epoxy resin system (Delta Tech S.p.A., Rifoglieto, Italy). The thickness of the CFRP plates used for the exterior faces is 0.5 mm. The skin/face density is 1172 kg/m [31]. The mechanical properties of the adopted CFRP prepreg are shown in Table 1.

Core materials were selected to cover a wide range of densities (Table 2), from 32 to 150 kg/m^3^, and structural architectures, enabling a systematic evaluation of their influence on the flexural behavior of CFRP sandwich specimens.

The material properties presented in Table 2 were used as reference parameters for material selection and comparative analysis of the investigated core materials. Polymeric cores include closed-cell polymer foams and a non-woven polymer core. ROHACELL^®^ 51 is a polymethacrylimide (PMI) foam with a nominal density of approximately 52 kg/m^3^, characterized by high specific stiffness and good thermal stability, making it suitable for aerospace sandwich applications [32]. AIREX C71.75, AIREX T92.100, and AIREX R82.150 are polyvinyl chloride (PVC)-based structural foams with nominal densities of approximately 75, 100, and 150 kg/m^3^, respectively [33]. These materials are commonly used in load-bearing sandwich structures due to their balanced shear strength, toughness, and processability. Soric XF [34], with a nominal density of approximately 62.5 kg/m^3^, is a non-woven polymer core designed to facilitate resin flow while providing structural support; its deformation behavior under bending differs from that of conventional closed-cell foams.

**Figure 1:**
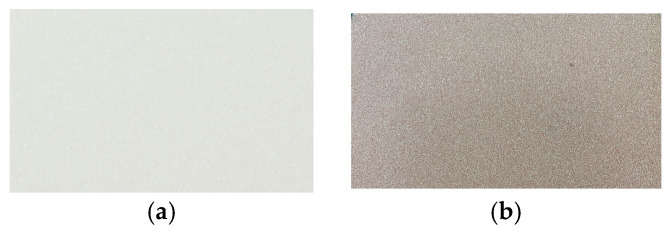

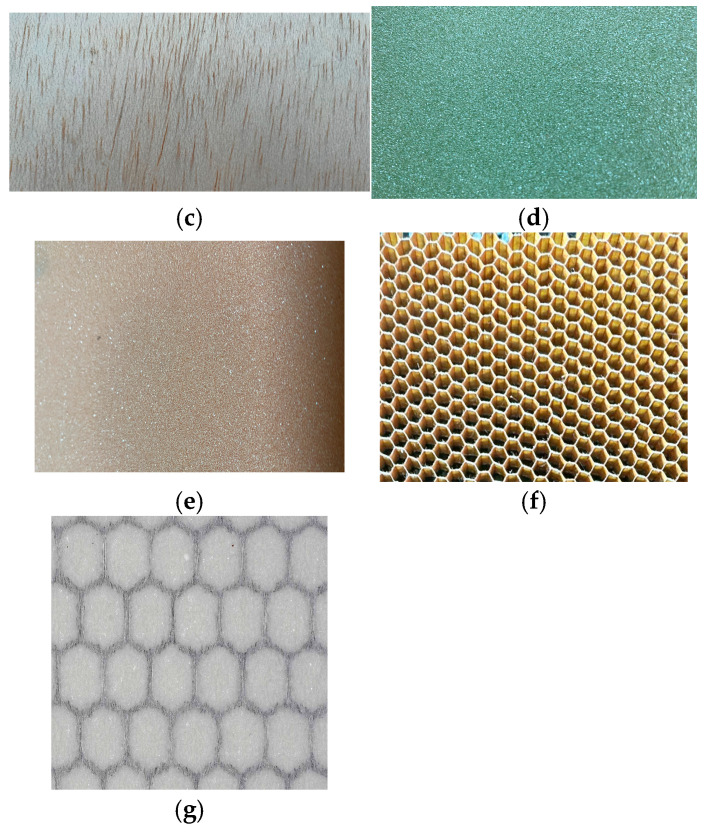
Core materials: (**a**) ROHACELL51 specimen; (**b**) AIREX T92.100 specimen; (**c**) balsa specimen; (**d**) AIREX R82.150 specimen; (**e**) AIREX C71.75 specimen; (**f**) NOMEX ECA_I specimen; (**g**) SORIC XF specimen.

The wooden core investigated in this study is end-grain balsa wood with a nominal density of approximately 100 kg/m^3^. Balsa cores are widely employed in sandwich structures due to their favorable stiffness-to-weight ratio and energy absorption capability, although their mechanical properties are strongly dependent on density and grain orientation. Another core type is represented by a NOMEX^®^ honeycomb core, based on aramid paper, with a nominal density of approximately 32 kg/m^3^ [35]. Figure 1 presents the core materials investigated in this study. The images highlight the different core architectures, including polymeric foams, balsa wood, and honeycomb structures.

### 2.2. Sample Manufacturing

**Figure 2:**
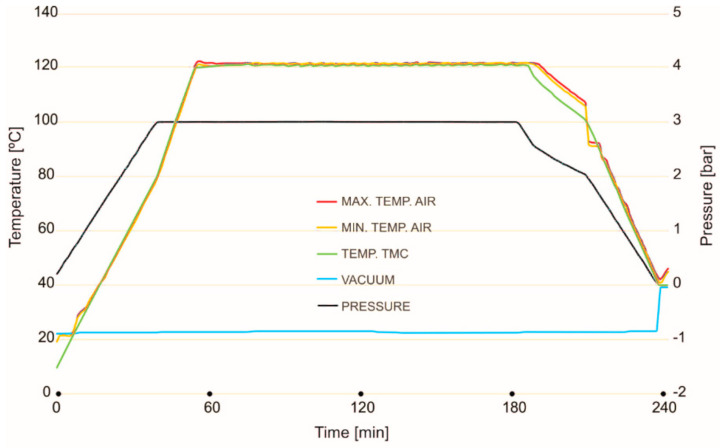
Autoclave curing cycle of sample manufacturing.

All sandwich specimens were fabricated using a prepreg-based layup process. Two layers of CFRP prepreg were applied to each side of the selected core material, forming symmetric face sheets. The assembled layups were vacuum-bagged between flat metal sheets to ensure uniform pressure distribution and thickness control. Curing was performed in an autoclave following the thermal cycle specified in Figure 2 and the parameters shown in Table 3.

**Figure 3:**
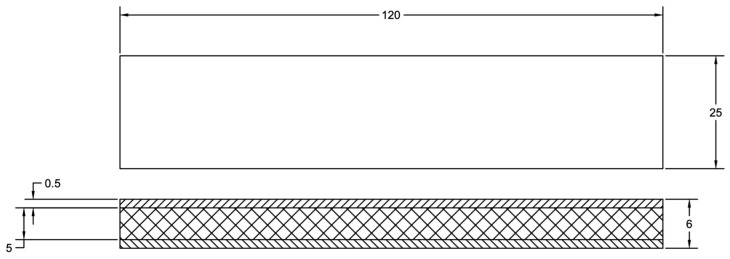
Specific dimensions. All the dimensions are in mm.

After curing, the panels were demolded and cut into test specimens according to the dimensional and geometric requirements specified in ASTM C393/C393M for sandwich beam flexural testing [36,37], as is shown in Figure 3.

The sandwich panel specimens were water-jet-cut, using an OptiMAX 60X machine (Romax, Bucharest, Romania), according to the dimensions specified in the ASTM C393/C393M standard. Particular attention was paid to mitigating cutting-induced damage associated with the initial piercing stage, during which the water-hammer effect may generate local delamination or skin–core debonding due to the high-pressure impact of the water jet. To prevent such defects, the piercing operation was performed outside the specimen geometry, within the surrounding sacrificial material, and the cutting path subsequently entered the contour of the specimen. This approach eliminated direct exposure of the specimen edges to the initial hydraulic shock. Following machining, the specimens were visually inspected, and no significant edge delamination, core crushing, or skin–core separation was observed. Particular attention was required for the balsa and Nomex honeycomb cores, as both are susceptible to water absorption during the cutting process, balsa due to its porous cellular wood structure, and Nomex due to its open-cell honeycomb geometry, which readily traps moisture at the cut edges. To eliminate the influence of residual moisture on the mechanical properties, all specimens were oven-dried (12 h at 35 deg C) prior to three-point bending testing. This conditioning step was applied uniformly across all core types (Airex, Rohacell, Nomex, and balsa) to ensure consistent baseline moisture conditions for valid comparison between core configurations.

**Figure 4:**
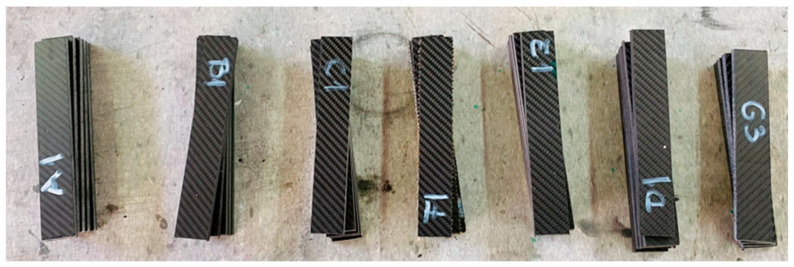
Manufactured samples: A (Rohacell 51), B (AIREX T92.100), C (balsa wood), D (AIREX R82.150), E (AIREX C71.75), F (Nomex), and G (Soric XF).

Five specimens were manufactured from each core material, and the resulting samples are shown in Figure 4.

### 2.3. Flexural Testing and Microscopy Investigations

Flexural tests were performed in accordance with ASTM C393/C393M to characterize the bending response of the CFRP sandwich specimens and to evaluate the influence of the core material on flexural stiffness, strength, and failure behavior. The tests were conducted under quasi-static loading conditions. Flexural tests were conducted using an INSTRON 336 series universal testing machine (Instron Co., High Wycombe, UK), providing controlled loading conditions and reliable acquisition of force–displacement data throughout the tests [38].

The specimens were extracted from different regions of the manufactured sandwich panel to minimize the influence of local material variability. Following water-jet cutting, all specimens were visually inspected and dimensionally verified before testing to ensure consistency within each group. For each core configuration, five specimens were tested under identical conditions. The reported values correspond to the arithmetic mean of the experimental results, while the standard deviation was calculated from the measured values of the replicate specimens.

**Figure 5:**
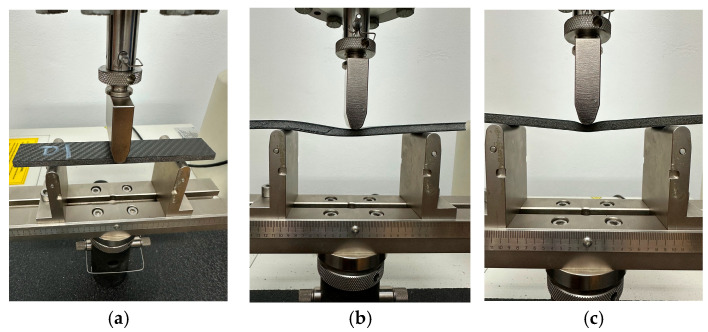

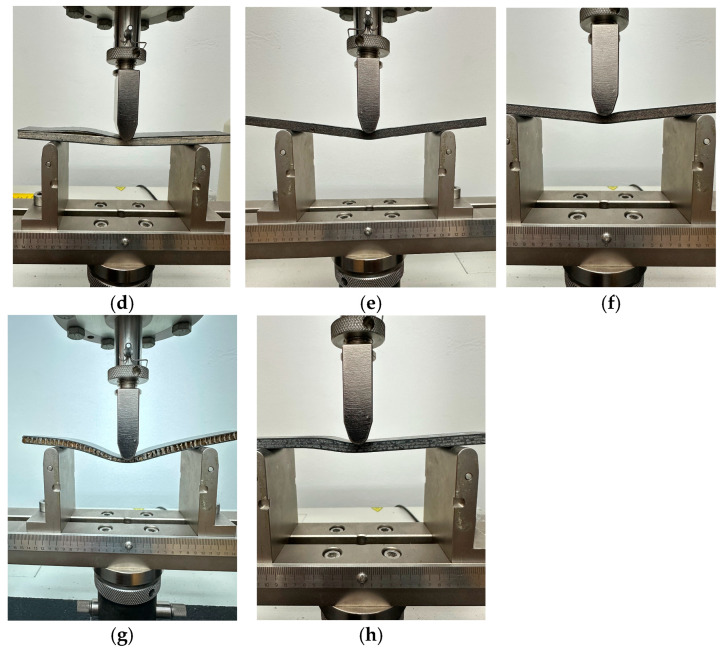
Three-point bending tests of CFRP sandwiches: (**a**) loading start; (**b**) ROHACELL51 specimen; (**c**) AIREX T92.100 specimen; (**d**) balsa specimen; (**e**) AIREX R82.150 specimen; (**f**) AIREX C71.75 specimen; (**g**) NOMEX ECA_I specimen; (**h**) SORIC XF specimen.

All specimens were tested using a three-point bending setup (Figure 5), with a constant support span selected according to the specimen thickness and the recommendations of the standard. Load was applied at the mid-span through a cylindrical loading nose, while the specimens were supported on two cylindrical supports to minimize stress concentrations. Three-point bending tests were performed using a fixture equipped with a 10 mm diameter loading nose and 10 mm diameter support rollers, with a support span of 102 mm. All tests were conducted at 21 °C using a constant crosshead speed of 4 mm/min.

The reported flexural strength and flexural modulus refer to the effective flexural response of the complete CFRP sandwich structure, as determined from three-point bending tests. Therefore, these values represent the structural behavior of the sandwich panel rather than the intrinsic mechanical properties of the constituent materials. The flexural modulus was obtained directly from the testing machine software based on the slope of the linear elastic region of the load–deflection curve, determined by linear regression within the 0.05–0.25% strain interval. The calculation followed the ASTM C393 testing configuration implemented in the software, using the recorded load, displacement, specimen span, width, and thickness as input parameters.

Microscopy investigations, after bending tests, were performed using an Optika C-B10 (Optika S.r.l., Bergamo, Italy), and the morphological structures of the samples were analyzed.

### 2.4. Comparative Analysis of Flexural Behavior and Maximum Efficiency of Sandwich Cores

The mechanical performance of sandwich composite structures is governed not only by their absolute stiffness but also by their efficiency in terms of mass. For lightweight engineering applications, the ratio between structural stiffness and mass represents a key design parameter, enabling objective comparison between different core materials and configurations. In the present study, the mass–stiffness relationship of sandwich specimens with identical face sheets and varying core materials is investigated based on three-point bending tests. The flexural response is evaluated using the load–displacement curves obtained experimentally. The flexural performance and mass efficiency of the investigated sandwich cores are compared to identify materials suitable for lightweight drone structures. By correlating flexural strength with specimen mass and core density, the strength-to-weight efficiency of each configuration is evaluated in relation to key UAV design requirements, including high stiffness, sufficient load-bearing capacity, and minimal structural mass. While denser cores generally exhibited higher absolute flexural strength, the ranking changes significantly when performance is normalized by specimen mass.

The present analysis does not aim to compare intrinsic material properties, but rather the structural efficiency of different sandwich configurations. Since all specimens share identical face sheets and geometrical parameters, the second moment of area remains constant. Therefore, the flexural modulus obtained experimentally represents an effective structural property that incorporates both bending and core shear contributions.

Consequently, the stiffness-to-weight efficiency can be assessed by normalizing the experimentally determined flexural modulus with respect to mass, enabling a direct comparison of the structural performance of each core configuration. The comparison is therefore performed at the structural level rather than at the material level, allowing the identification of core configurations that maximize bending performance per unit mass under identical geometric and loading conditions.

The specific stiffness index was selected because it directly relates the measured flexural stiffness to the actual mass of the tested sandwich panel. While specific modulus values are commonly normalized using material density, the present approach is more representative for lightweight UAV structures, where the overall structural mass is a primary design constraint. Since all specimens possessed identical external dimensions, the use of specimen mass provides an equivalent basis for comparison while directly reflecting the weight-saving potential of each core configuration.

In order to assess the structural efficiency of the investigated sandwich configurations, the specific stiffness (stiffness-to-weight ratio) was evaluated based on the flexural response obtained from three-point bending tests. The bending stiffness of each specimen is expressed as (1):

(1) $$D=E_{f}I$$

where $E_{f}$ is the experimentally determined flexural modulus and I is the second moment of area of the cross-section. The specific stiffness was defined as (2):

(2) $$\frac{D}{m}=\frac{E_{f}I}{m}$$

where m is the mass of the specimen. Since all tested configurations share identical geometric parameters and face sheets, the second moment of area I remains constant.

### 2.5. Statistical Analysis

Preliminary tests were performed, focused on testing data normality and equal variances. Using the Ryan–Joiner test and Levente test, normality and homogeneity of data were not found. The p parameter was lower than 0.05 for both tests. Thus, one-way analysis of variance (ANOVA) with Box–Cox transformation with an optimal lambda coefficient was performed for both targets, flexural strength and flexural modulus. The optimal lambda value was 0.024 in the case of the flexural stress study, and 0.435 in the case of the flexural modulus study. The optimal lambda produced the best fitting transformation. The ANOVA was performed for all seven categories of core sandwiches, focused on core parameters. Tukey’s Honestly Significant Difference test was performed to highlight significant differences between groups. The statistical analysis was performed using Minitab 19 software (Minitab LLC, State College, PA, USA).

## 3. Results

The results are grouped in the following sections: flexural properties of samples, statistical results, microscopy investigations, and application selection of sandwich architectures for drone structures.

### 3.1. Flexural Properties of Sandwich Specimens

**Figure 6:**
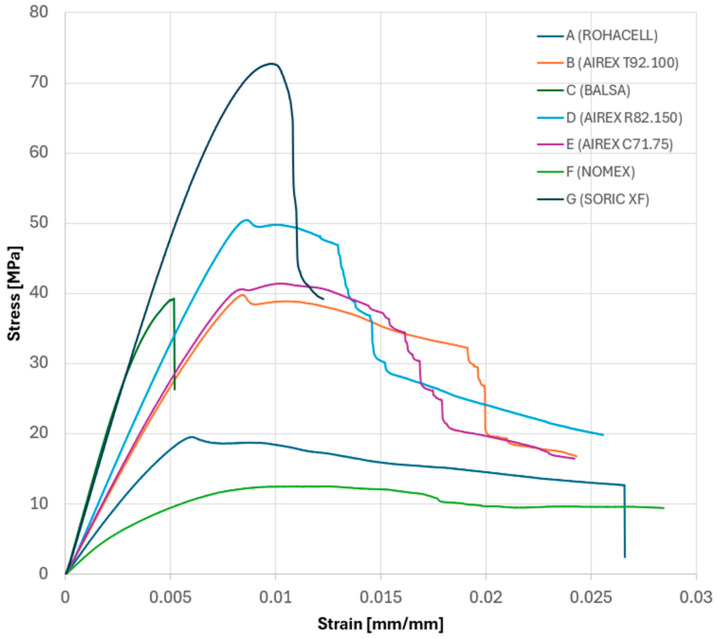
Flexural stress–strain experimental curves.

The flexural stress–strain response presented in this section is based on the experimental curves obtained from four nominally identical specimens for each core material (Figure 6).

**Figure 7:**
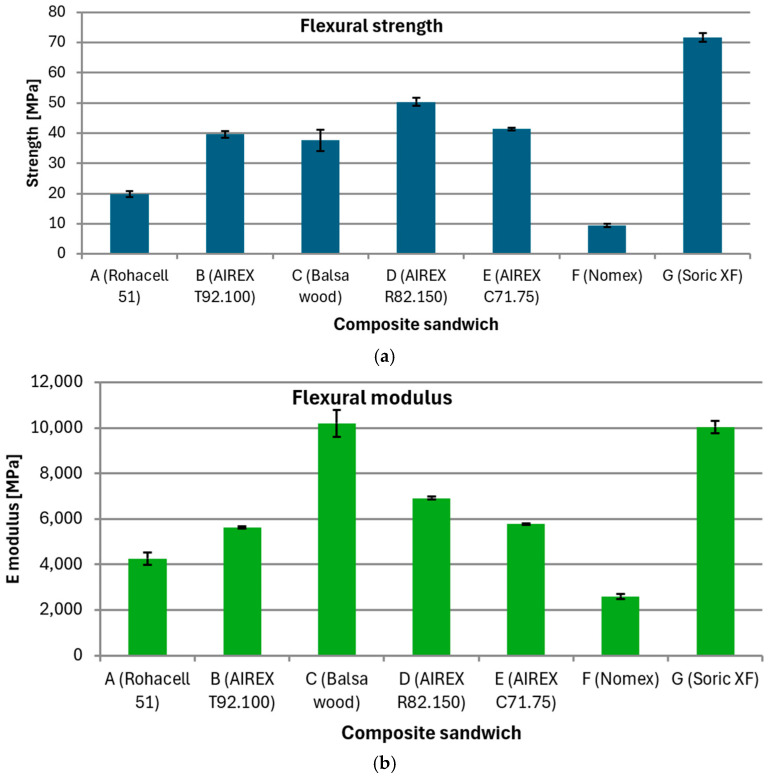
Mechanical properties of composite samples: (**a**) flexural strength; (**b**) flexural modulus.

The individual curves are reported to illustrate the repeatability of the flexural behavior and to capture the variability associated with each sandwich configuration. Representative trends and differences between core types are discussed based on the collective response of the tested specimens. The flexural modulus was determined through linear regression applied to the stress–strain response within the linear elastic region (0.05–0.25% strain), consistent with established characterization practice for foam-core and honeycomb-core sandwich structures. The mean values and corresponding sample standard deviations are summarized in Table 4, and a comparative study is presented in Figure 7.

The results demonstrate a pronounced variation in flexural performance across the investigated core configurations, highlighting the dominant role of core architecture in sandwich behavior. Soric^®^ exhibited the highest mean flexural stress (71.66 MPa) combined with an exceptionally low coefficient of variation (1.88%), indicating both superior load-bearing capacity and outstanding repeatability. This response is attributed to its uniform, engineered structure, which promotes efficient and homogeneous load transfer at the skin–core interface.

The relatively low flexural strength values observed in some configurations can be attributed to the lightweight nature of the investigated core materials. Although the CFRP face sheets exhibit high mechanical properties, the overall flexural response of sandwich structures is often governed by core-related failure mechanisms such as shear deformation, local crushing, and skin–core debonding. Consequently, the measured flexural strength reflects the behavior of the complete sandwich structure rather than the intrinsic strength of the CFRP laminates.

In contrast, the Nomex^®^ honeycomb core showed the lowest flexural stress (9.42 MPa), confirming the limited contribution of open-cell cores under localized bending loads. As widely reported, the structural response in such configurations is largely governed by the face sheets, with the core primarily stabilizing the geometry rather than contributing to stiffness.

Among the foam-based materials, AIREX R82.150 achieved the highest flexural stress (50.31 MPa) and modulus (6917.41 MPa), consistent with its higher density and improved load distribution capability. AIREX T92.100 and C71.75 exhibited comparable stress levels, although C71.75 showed reduced variability, indicating more consistent structural performance. Rohacell^®^ displayed significantly lower stress and modulus values, reflecting its reduced stiffness contribution within the sandwich system, in agreement with reported PMI core behavior [2,5].

Balsa wood presented a distinct response, combining high stiffness (10,204.65 MPa) with moderate strength (37.59 MPa), but at the cost of substantial variability (CV = 9.17%). This scattering is a direct consequence of its anisotropic and heterogeneous microstructure, which leads to inconsistent load transfer and failure initiation. In contrast, all synthetic cores exhibited low dispersion (CV < 10%), reinforcing the reliability advantages of engineered materials.

From a stiffness perspective, Soric^®^ and balsa clearly outperform foam cores, demonstrating the strong influence of core architecture on bending rigidity. Given that all specimens share identical face sheets, these differences can be directly attributed to the core response. Conversely, Nomex^®^ exhibited the lowest modulus (2597.53 MPa), consistent with its low through-thickness stiffness and deformation-controlled behavior.

Although Soric XF has a lower nominal density than Airex R82.150, its superior flexural strength indicates that the bending response of the sandwich panel is governed not only by core density but also by the core architecture and its interaction with the CFRP face sheets. The resin-infused fibrous network of Soric XF promotes efficient load transfer and delays localized damage, resulting in higher load-carrying capacity under bending. In contrast, the cellular structure of Airex R82.150 primarily resists deformation through cell wall compression and shear, leading to earlier stiffness degradation despite its higher density.

Overall, the results underline a clear trade-off between stiffness, strength, and reliability. While balsa maximizes stiffness, its variability limits its applicability in controlled structural designs. In contrast, Soric^®^ and AIREX R82.150 provide the most balanced performance, combining high mechanical response with consistent behavior, making them more suitable for reliable lightweight sandwich structures.

The flexural performance of the investigated sandwich specimens, tested under three-point bending conditions, is summarized in Table 5.

The comparison includes key parameters such as peak stress, initial stiffness, post-peak response, and the dominant failure mechanisms associated with each core material. It should be noted that the reported values reflect the global structural behavior of the sandwich configurations, as the response is governed by the interaction between the face sheets and core. The results highlight significant differences in both load-bearing capacity and failure modes, allowing the identification of distinct regimes of behavior, ranging from face-dominated brittle failure to core-governed progressive collapse.

This section interprets the flexural test results obtained from the stress–strain curves and associated parameters, including flexural strength, strain at tensile strength, and flexural modulus. For each core, the five specimens exhibit a consistent mechanical response characterized by an initial linear elastic region followed by a nonlinear regime leading to peak stress and subsequent softening. Such behavior is consistent with previous observations on polymeric and scored foam sandwich beams subjected to bending loads. The relatively low coefficients of variation observed for flexural strength and modulus indicate good repeatability and satisfactory material uniformity within each core type, aligning with ASTM C393 recommendations for sandwich beam testing. Variations in peak stress and post-peak response reflect the influence of core morphology, density, and architecture on load transfer and deformation mechanisms, as recently shown for various core designs including grid-scored foams and additive manufacturing-based cores. Strain at tensile strength provides insight into deformability and energy absorption capacity. Higher strain values suggest increased ductility, while lower values indicate stiffer response—an effect also noted in multimaterial sandwich beams with curved cores and differing densities. These trends illustrate the inherent trade-offs between stiffness, strength, and flexibility that must be balanced in lightweight structural design.

Overall, the observed flexural performance confirms that core material selection critically affects the stiffness, strength, and failure characteristics of sandwich structures, echoing recent findings on advanced core designs and biomimetic structural optimization strategies. Accordingly, the ratio $E_{f}/m$ was used to evaluate the relative structural efficiency of the different core configurations. The calculated values are summarized in Table 6, which presents the flexural modulus, specimen mass, and resulting specific stiffness for each configuration. The table enables a direct comparison of stiffness efficiency, highlighting the influence of core architecture on the overall bending performance of the sandwich structures.

As shown in Table 6, balsa and Soric^®^ cores exhibit the highest specific stiffness values, indicating superior stiffness-to-weight efficiency. While balsa achieves the highest value overall, its previously observed variability must be considered in practical applications. Soric^®^, in contrast, combines high specific stiffness with excellent repeatability, making it a more reliable structural solution [39,40].

Among the foam-based cores, AIREX R82.150 demonstrates the best performance, followed by AIREX C71.75 and T92.100, which exhibit comparable efficiency. Rohacell^®^ shows lower specific stiffness, reflecting its reduced contribution to global bending rigidity. The Nomex^®^ honeycomb core presents the lowest stiffness-to-weight ratio, consistent with its low through-thickness stiffness and deformation-dominated behavior. Overall, the results confirm that the stiffness efficiency of sandwich structures is strongly dependent on core architecture, and that optimal material selection requires balancing stiffness, weight, and variability.

The results presented in Table 6 highlight significant differences in stiffness–mass performance among the investigated core materials. Balsa exhibits the highest flexural stiffness and specific stiffness, indicating superior rigidity; however, this must be considered alongside its previously observed brittle failure behavior. Among the synthetic cores, SORIC XF and AIREX R82.150 provide the best balance between stiffness and mass, showing high specific stiffness values combined with stable mechanical response. AIREX T92.100 and C71.75 demonstrate moderate performance with good consistency, while ROHACELL shows lower stiffness despite its relatively low mass. NOMEX exhibits the lowest stiffness, although its lightweight nature and progressive deformation behavior may still be advantageous in applications where energy absorption is prioritized over rigidity.

**Figure 8:**
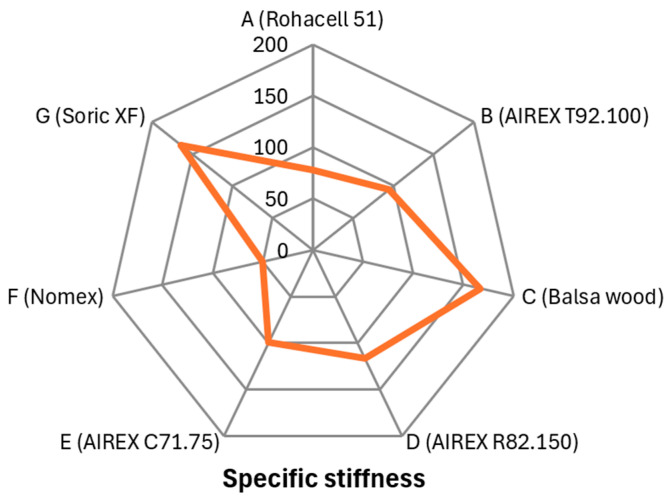
Specific stiffness diagram.

The mass–stiffness diagram (Figure 8) illustrates the distribution of the investigated sandwich core materials in terms of flexural performance and weight efficiency. Each point represents a specific core configuration, positioned according to its measured mass and experimentally determined flexural stiffness. A clear differentiation between material types can be observed. Balsa occupies the upper region of the plot, exhibiting the highest stiffness, while NOMEX is located in the lower-left region, indicating low stiffness but reduced mass. Synthetic foam cores such as AIREX variants and ROHACELL are grouped in an intermediate region, with SORIC XF and AIREX R82.150 showing the most favorable balance between stiffness and mass. Overall, the figure highlights the trade-off between structural rigidity and weight, allowing the identification of core materials that provide optimal performance for lightweight sandwich structures to be used in UAV structures.

### 3.2. Statistical Results

The results of one-way ANOVA with Box–Cox transformation are presented in Table 7 for both responses, flexural strength and flexural modulus. The ANOVA results are significant because the *p*-value is lower than 0.001 in both cases.

The coefficients of determination, including R-sq, R-sq(adj), and R-sq(pred), were determined with values higher than 99%, as is shown in Table 8. Thus, the models of flexural strength and flexural modulus fit the actual data.

**Figure 9:**
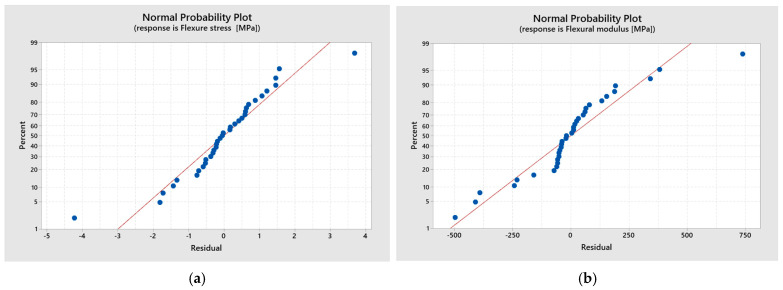
Residual normality plot for: (**a**) flexural strength; (**b**) flexural modulus.

Normally distributed residuals were found for both cases, as is shown in Figure 9.

Tukey’s test showed that groups sharing the same letter were not significantly different, and groups with different letters were significantly different, as is shown in Table 9 and Table 10.

Thus, it was found, for flexural strength, that the sandwiches based on Airex C71.75 and AIREX T92.100 are not significantly different. Also, samples B (AIREX T92.100) and C (balsa wood) are not significantly different from the point of view of flexural strength (Table 9). The Tukey’s pairwise comparison results for the flexural modulus show that for C (balsa wood) and G (Soric XF) and for E (AIREX C71.75) and B (AIREX T92.100), groups sharing the same letter are not significantly different (Table 10).

**Figure 10:**
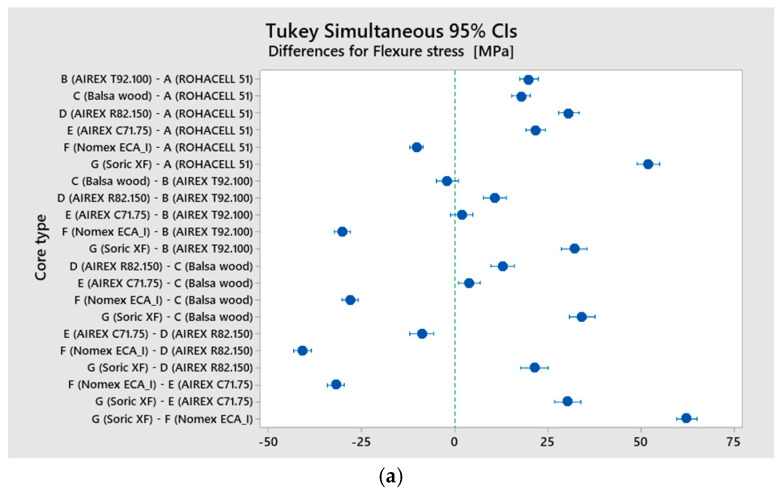

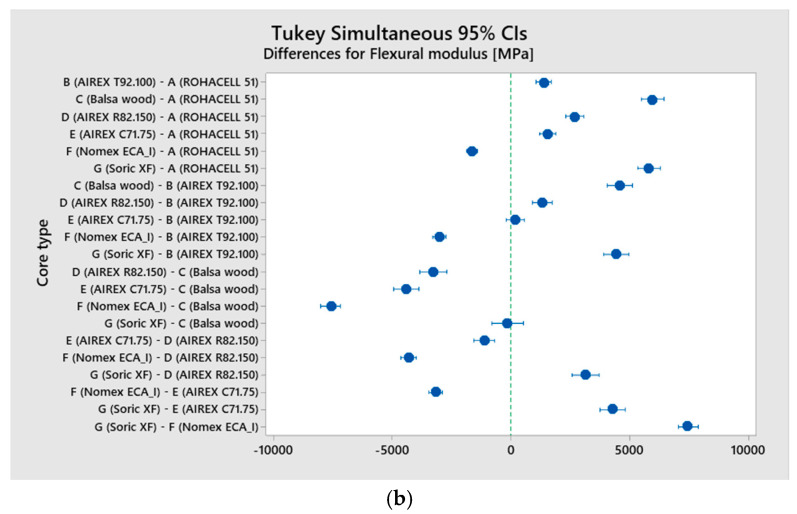
Tukey’s pairwise comparison at 95% confidence interval for: (**a**) flexural strength; (**b**) flexural modulus.

From the graphical results of the Tukey test it was found that if an interval does not contain zero, the corresponding mean is significantly different. It was graphically validated that the mean intervals of Airex C71.75 and AIREX T92.100 and of AIREX T92.100 and balsa wood for flexural strength, as well as of balsa wood and Soric XF and of AIREX C71.75 and AIREX T92.100 for flexural modulus, were similar (Figure 10a,b).

**Figure 11:**
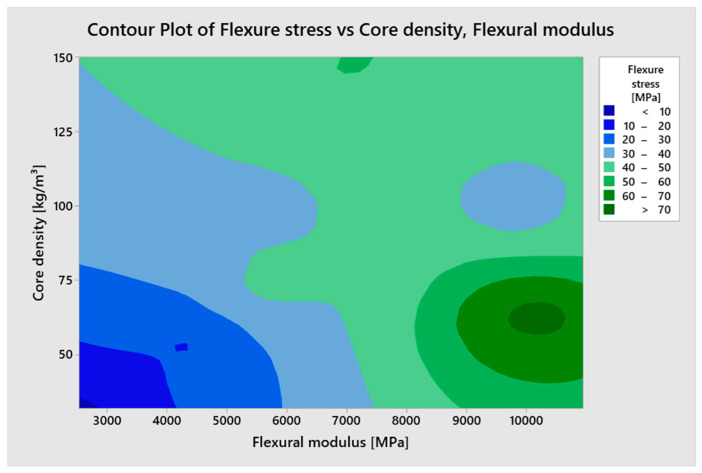
Contour plot of flexural stress versus core density and flexural modulus.

A contour plot of flexural stress versus core density and flexural modulus is shown in Figure 11. It is useful to understand how changes in core density and flexural modulus influence the flexural stress of the sandwich. At lower modulus values (<4500 MPa), the stress is mostly in the blue region (<30 MPa). At higher modulus values (8500–10,500 MPa), the stress shifts to the green and dark green regions (>50 MPa). This indicates that materials with higher stiffness exhibit greater resistance to bending loads. It was found that around 40–70 kg/m^3^, especially with high modulus values, the highest stress levels are observed (>70 MPa). The relationship between density and flexural stress is not linear. Thus, moderate-to-low-density cores combined with high modulus produce optimum stress performance. Also, very high core density does not necessarily improve flexural stress.

**Figure 12:**
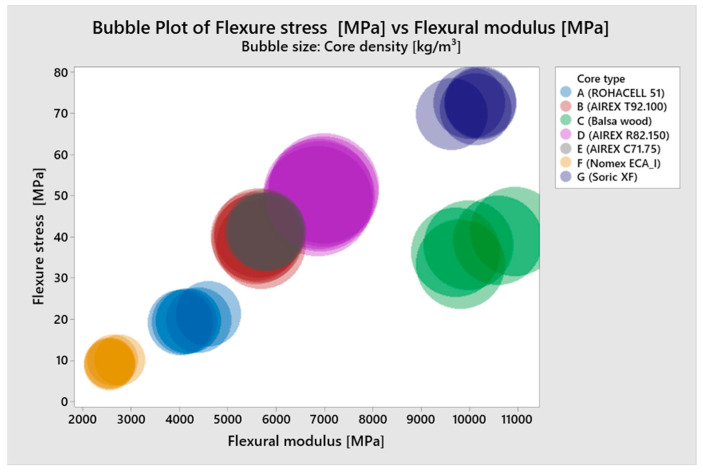
Bubble plot representation of flexural stress versus flexural modulus.

A bubble plot representation of flexural stress versus flexural modulus, considering all types of sandwiches, is shown in Figure 12. The lowest value for the flexural modulus was found for F (NOMEX ECA_I), which has the lowest density. Samples G (Soric XF) and C (balsa wood) had the highest values for the flexural modulus, but their densities are different. The main disadvantages of the balsa core compared to the Soric XF core are higher weight, moisture sensitivity, lower flexibility for complex shapes, and natural variability. In conclusion, Soric XF is preferred for lightweight structures and easy-processing composite panels used for drones [41,42].

### 3.3. Microscopic Investigations of Core Damage and Fracture Mechanisms

Microscopic investigation of the fractured sandwich specimens was conducted to identify damage mechanisms and failure modes at the core and skin–core interface following flexural testing. The analysis focused on assessing cell deformation, interfacial integrity, and local damage features [43] that contribute to the global mechanical response of the sandwich structures.

- A (ROHACELL 51)

**Figure 13:**
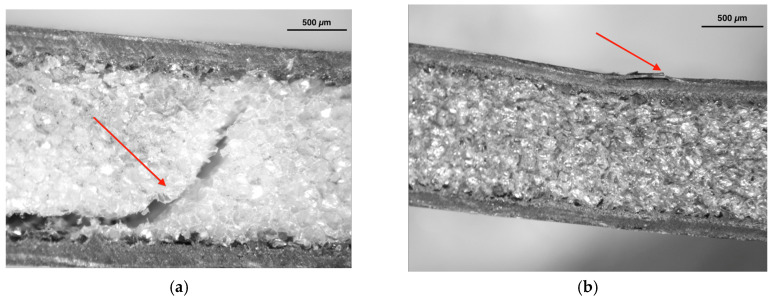

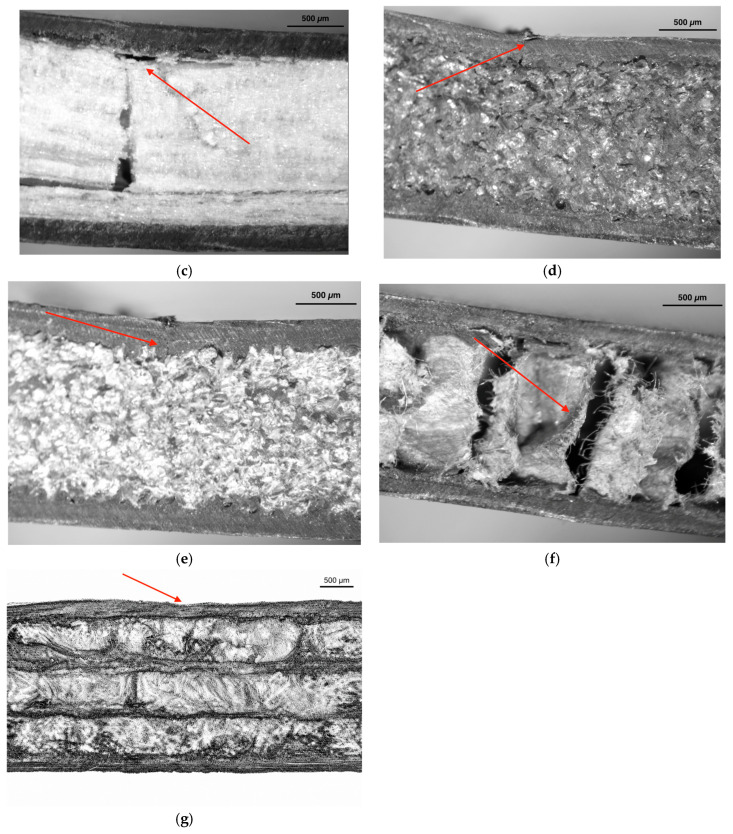
Microscopic images of the fracture zone of the CFRP sandwich specimens with the following cores: (**a**) A (ROHACELL51) specimen; (**b**) B (AIREX T92.100) specimen; (**c**) C (balsa) specimen; (**d**) D (AIREX R82.150) specimen; (**e**) E (AIREX C71.75) specimen; (**f**) F (NOMEX ECA_I) specimen; (**g**) G (SORIC XF) specimen.

The Rohacell foam core exhibits localized shear cracking and cell wall rupture, concentrated near the mid-thickness of the core (Figure 13a). The fracture path appears relatively clean and planar, indicating a brittle shear-dominated failure rather than progressive crushing. Limited plastic deformation of the foam cells is observed, suggesting that failure occurred once the shear stress exceeded the intrinsic strength of the closed-cell polymer structure.

The observed failure mode indicates that the skin–core interface remained structurally intact throughout most of the loading process, confirming efficient stress transfer between the CFRP face sheets and the PMI core. Consequently, the flexural response was governed by the intrinsic shear capacity of the Rohacell foam rather than by interfacial debonding (arrow indication). This explains the relatively stable linear elastic response followed by an abrupt loss of load-carrying capacity once the critical shear stress within the core was reached.

- B (AIREX T92.100), D (AIREX R82.150) and E (AIREX C71.75)

The AIREX-based cores, Figure 13b,d,e, exhibit a ductile, shear-dominated failure mechanism characterized by progressive cell wall collapse and plastic deformation across the core thickness. Damage is distributed over a relatively wide region, with no sharp or brittle fracture plane, indicating effective energy absorption prior to failure. Localized crushing beneath the loading region and gradual shear band formation suggest that flexural failure is governed by core shear yielding rather than abrupt cracking. This response is consistent with the viscoelastic nature of AIREX foams and supports their suitability for drone sandwich structures where damage tolerance and gradual failure are preferred over brittle collapse. In the case of the T92.100 core (Figure 13b), damage initiation was accompanied by localized debonding (indicated by the arrow) of the upper CFRP face sheet, while the PET foam remained largely intact, indicating that the onset of failure was controlled by interfacial stress concentrations rather than extensive core fracture.

- C (Balsa)

For the balsa core specimen, the microscopic analysis reveals a localized initiation of delamination at the skin–core interface, Figure 13c, propagating along the fiber direction of the wooden core. The arrows indicate the regions where interfacial separation begins at the boundary between the CFRP skin and the balsa core, driven by flexural-induced shear and tensile stresses. This damage pattern reflects the anisotropic nature of the balsa structure, which promotes crack propagation along the wood fibers rather than through the core thickness. The gradual onset of delamination suggests a progressive interfacial failure mechanism, relevant for assessing the damage tolerance of wood-core sandwich structures. Furthermore, a vertical crack within the balsa core is observed (arrow indication), indicating local splitting of the wood cellular structure prior to extensive interfacial separation. This combined failure mode, involving core splitting followed by skin–core delamination, reflects the orthotropic nature of balsa and explains its high initial flexural stiffness together with the reduced damage tolerance observed after crack initiation. Once the interface integrity is compromised, the efficiency of shear load transfer between the CFRP face sheets and the core decreases rapidly, leading to progressive degradation of the sandwich structural response.

- D (Airex R82.150)

The AIREX R82.150 core exhibited a stable damage mechanism characterized by localized cell wall deformation accompanied by limited skin–core debonding, as indicated in Figure 13d. Unlike the brittle crack propagation observed in Rohacell or the interfacial delamination identified in the balsa specimens, no continuous fracture path developed through the foam thickness. Instead, the closed-cell PEI structure promoted gradual deformation and distributed shear stresses over a larger volume of the core, delaying catastrophic failure. The limited extent of interfacial damage confirms efficient load transfer between the CFRP face sheets and the core throughout most of the loading process. This progressive damage evolution explains the favorable balance between flexural stiffness, strength, and weight efficiency obtained for the R82 configuration, making it the most suitable core material among the investigated candidates for lightweight UAV sandwich structures.

- E (AIREX C71.75)

The AIREX C71.75 core (Figure 13e) exhibited localized cell wall deformation beneath the compressed CFRP face sheet, without the formation of a continuous crack or significant skin–core debonding. Damage remained confined to the upper region of the PVC foam, indicating that failure was governed by progressive cell collapse rather than brittle fracture. This behavior allowed the core to maintain effective shear load transfer during bending, resulting in a gradual reduction in stiffness instead of abrupt structural failure.

- F (NOMEX ECA_I)

The NOMEX honeycomb core exhibited a collapse-dominated failure mechanism, characterized by buckling and progressive folding of the honeycomb cell walls under flexural loading, as shown in Figure 13f. The micrograph reveals severe local instability and crushing of the cellular architecture, leading to a significant reduction in the core’s ability to transfer shear loads between the CFRP face sheets. Unlike the foam cores, failure was governed by structural instability of the honeycomb rather than bulk material fracture or extensive interfacial debonding. This instability-driven mechanism explains the low flexural strength and reduced structural efficiency observed for the NOMEX-based sandwich panels.

- G (Soric XF)

For the Soric XF core micrograph, the failure mechanism is characterized by localized deformation and cell compaction without the formation of distinct cracks (Figure 13g). The cellular structure exhibits progressive collapse and densification, particularly in regions subjected to maximum shear stresses during flexural loading, while maintaining the overall integrity of the core architecture. The absence of sharp fracture planes or extensive skin–core debonding indicates a highly damage-tolerant response, allowing stresses to be redistributed progressively rather than released through catastrophic failure. Although Soric XF has a relatively low nominal dry density, its three-dimensional flow-media architecture retains a significant amount of epoxy resin during the autoclave curing process. The resulting resin-rich network increases the effective density of the manufactured sandwich panel and enhances the mechanical interlocking and shear load transfer between the CFRP face sheets. This synergistic effect explains the superior flexural strength achieved by the Soric XF specimens. However, the increased resin uptake also resulted in the highest specimen mass among all investigated configurations, reducing its weight efficiency compared with the Airex R82 core, which provided the best overall balance between flexural performance and structural weight for UAV applications.

### 3.4. Application-Driven Selection of Sandwich Architectures for Drone Structures

**Figure 14:**
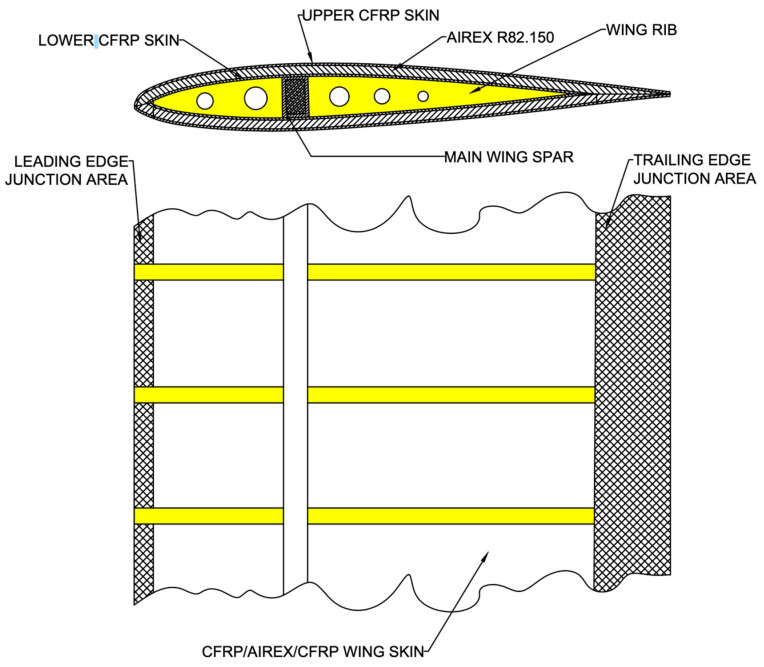
Schematic representation of a UAV wing with sandwich skin.

A schematic representation of the proposed solution consisting of sandwich skin features is presented in Figure 14. This solution can be used for large unmanned aerial vehicle structures, where wingspans may approach 10 m and achieving a high strength-to-weight ratio is a critical design requirement due to its direct impact on payload capacity, flight endurance, and structural reliability. In this context, CFRP-based sandwich constructions are widely adopted in aerospace and UAV applications because they provide high flexural stiffness and load-bearing capability at minimal mass compared to monolithic laminates. The mechanical response of such structures is strongly governed by the core material, which controls shear load transfer, energy absorption, and damage evolution under bending-dominated loading conditions. Environmental factors such as temperature and humidity were not considered in the present investigation, as the primary objective was to isolate and quantify the influence of core configuration on the flexural behavior of CFRP sandwich panels under identical testing conditions. Introducing environmental aging variables would have added additional sources of variability, potentially masking the specific contribution of the core material to the measured mechanical response.

The present results indicate that sandwich architectures combining CFRP prepreg skins with damage-tolerant polymeric cores, such as Soric XF and high-performance PMI-based foams, offer the most favorable mass-normalized flexural performance. The Soric XF core, in particular, exhibits a deformation-dominated failure mechanism characterized by progressive cell compaction and effective stress redistribution, which delays structural collapse and avoids brittle fracture. Such behavior is especially advantageous for large-span UAV wings and control surfaces, where gradual damage progression and residual load-carrying capacity are preferred to enhance operational safety.

High-density PMI and advanced PVC foam cores (e.g., AIREX R-series) also demonstrate competitive specific performance and stable shear-dominated failure, making them suitable candidates for primary load-bearing regions such as wing roots, spar webs, and fuselage frames, where stiffness requirements are more stringent. In contrast, low-density honeycomb cores, such as NOMEX, may suffer from premature cell wall buckling and core collapse under out-of-plane bending, leading to an early loss of structural integrity in thick sandwich sections. NOMEX honeycomb structures may, however, be used in secondary structures. Natural balsa cores provide a favorable compromise between mass efficiency and sustainability; however, their intrinsic anisotropy can promote interfacial delamination along the wood fiber direction, suggesting their use should be limited to secondary or locally reinforced UAV structures. Overall, for large UAV platforms [44], the optimal sandwich architecture consists of CFRP prepreg skins combined with medium-density, polymeric cores that exhibit progressive failure behavior, prioritizing specific strength and damage tolerance [45] over minimum density alone.

## 4. Conclusions

This study investigated the flexural behavior, mass efficiency, and fracture mechanisms of CFRP sandwich structures manufactured from prepreg skins combined with different lightweight core materials, with the aim of identifying optimal architectures for large unmanned aerial vehicle structures. Three-point bending tests performed in accordance with ASTM C393, together with microscopic fracture analysis, enabled a comprehensive assessment of mechanical performance. The following conclusions have been drawn.

- Among the evaluated materials, Soric XF with 62.5 kg/m^3^ density exhibited the highest flexural strength (71.66 MPa), a 10,039 MPa flexural modulus and specific load-bearing capacity; owing to its progressive deformation and damage-tolerant failure mechanism, it had the best mechanical properties. PMI-based foam cores also demonstrated stable and repeatable mechanical behavior, whereas Nomex honeycomb low-density cores exhibited significantly lower flexural performance due to premature cell buckling. Although balsa offered an attractive sustainable alternative with favorable stiffness-to-weight characteristics, interfacial delamination observed during microscopic examination may restrict its use.
- The fracture analysis confirmed that superior mechanical performance is closely associated with progressive, damage-tolerant failure mechanisms, while brittle cracking, interfacial debonding, and localized core collapse lead to premature structural degradation. The statistical evaluation further validated the significant influence of core architecture on both flexural strength and flexural modulus, providing quantitative support for selecting medium-density polymeric foam cores for applications requiring consistent and reliable structural performance.
- Overall, the results indicate that the design of CFRP sandwich structures for large UAV wings should prioritize specific flexural performance, progressive damage evolution, and structural reliability rather than minimum density alone. The integration of mechanical testing, microscopic characterization, and statistical analysis presented in this work provides a comprehensive framework for selecting optimized sandwich core materials for bending-dominated aerospace structures.
- Although the present investigation was limited to quasi-static three-point bending, the findings establish a solid basis for future studies addressing fatigue, impact resistance, vibration, and long-term durability under representative operational conditions. Such investigations will further support the development of lightweight, damage-tolerant sandwich structures capable of meeting the increasingly demanding performance requirements of next-generation unmanned aerial vehicles.

## Figures and Tables

**Table 1:** Skin material properties [31].

Property	Value	Test Standard
Tensile Strength (0°/Warp)	675 MPa	ASTM D3039
Tensile Strength (90°/Weft)	654 MPa	ASTM D3039
Young’s Modulus, (E_1) (0°)	54.0–56.7 GPa	ASTM D3039
Young’s Modulus, (E_2) (90°)	55.0–56.7 GPa	ASTM D3039
In-Plane Shear Modulus, (G_{12})	4.2–6.06 GPa	ASTM D3518
Poisson’s Ratio, (\nu_{12})	0.04	ASTM D3039
Compressive Strength (0°)	570 MPa	ASTM D6641

**Table 2:** Core material properties [32,33,34,35].

Core Material	Structure Type	Core Density (kg/m^3^)	Flexural Strength [MPa]	Flexural Modulus [MPa]
ROHACELL 51	PMI, foam	52	1.25	75
AIREX T92.100	PET, foam	100	6	210
Balsa	Balsa, wood	100	10	1200
AIREX R82.150	PEI, foam	150	15	700
AIREX C71.75	PVC, foam	75	3.5	150
NOMEX ECA-I	ECA_I 4.8–32, honeycomb	32	0.5	100
Soric XF	Polyester, foam	62.5	1	35

**Table 3:** CFRP sandwich curing parameters.

Parameter	Value
Initial temperature	25 °C
Heating rate	1.6 °C/min
Maximum air temperature	122 °C
Tool/laminate temperature (TMC)	120 °C
Applied pressure	3 bar
Vacuum level	−0.85 bar
Cooling stage	Controlled cooling
Final temperature	25 °C
Total cycle duration	240 min

**Table 4:** Mean flexural stress and flexural modulus for sandwich samples (n = 5).

Sandwich	Mean Stress [MPa]	SD [MPa]	CV [%]	Mean Modulus [MPa]	SD [MPa]	CV [%]
A (Rohacell 51)	19.77	0.99	5.02	4254.39	259.45	6.10
B (AIREX T92.100)	39.60	1.11	2.79	5621.58	46.28	0.82
C (Balsa wood)	37.59	3.45	9.17	10,204.65	599.38	5.87
D (AIREX R82.150)	50.31	1.34	2.67	6917.41	62.95	0.91
E (AIREX C71.75)	41.38	0.44	1.06	5777.27	31.84	0.55
F (Nomex)	9.42	0.51	5.40	2597.53	105.10	4.05
G (Soric XF)	71.66	1.35	1.88	10,039.25	283.21	2.82

**Table 5:** Comparison between sandwiches.

Sandwich	Initial Stiffness	Post-Peak Behavior	Dominant Failure Mechanism	Structural Assessment
A (Rohacell 51)	Low	Smooth post-peak decay	Core-dominated shear failure	Stable but low load capacity
B (AIREX T92.100)	Moderate	Gradual softening	Core shear and compressive crushing	Stable, slightly lower performance
C (Balsa wood)	High	Sudden early failure	Brittle core shear/crack propagation	High stiffness, poor reliability
D (AIREX R82.150)	High	Plateau + gradual decay	Core shear and progressive crushing	Best overall performance (balanced)
E (AIREX C71.75)	Moderate	Smooth degradation	Core shear + localized crushing	Good stability, moderate strength
F (Nomex)	Very low	Progressive collapse	Honeycomb cell buckling and collapse	Excellent energy absorption, very low strength
G (Soric XF)	Very high	Abrupt drop (brittle)	Face-sheet failure (tension/compression) or local buckling	Highest strength, low damage tolerance

**Table 6:** Specific stiffness of sandwiches.

Sandwich	Mean Flexural Modulus (E_f_) [MPa]	Mass of Sandwich [g]	Specific Stiffness (E_f_/m) [MPa/g]
A (Rohacell 51)	4254.39	54.50	78.06
B (AIREX T92.100)	5621.58	59.50	94.48
C (Balsa wood)	10,204.65	61.30	166.47
D (AIREX R82.150)	6917.41	59.50	116.26
E (AIREX C71.75)	5777.27	58.40	98.93
F (Nomex)	2597.53	51.60	50.34
G (Soric XF)	10,039.25	61.20	164.04

**Table 7:** Analysis of variance of transformation response for flexural strength and flexural modulus.

	Source	DF	Adj SS	Adj MS	F-Value	*p*-Value
Flexural strength	Core type	6	92.8462	15.4744	1126.99	<0.001
	Error	28	0.3845	0.0137		
	Total	34	93.2306			
Flexural modulus	Core type	6	6.95436	1.15906	1053.25	<0.001
	Error	28	0.03081	0.00110		
	Total	34	6.98517			

**Table 8:** Model summary of transformation response for flexural strength and flexural modulus.

Response Factor	R-sq	R-sq (adj)	R-sq (pred)
Flexural strength	99.59%	99.50%	99.36%
Flexural modulus	99.56%	99.46%	99.31%

**Table 9:** Flexural strength Tukey test results at 95% confidence.

Core Type	N	Mean	Grouping					
G (Soric XF)	5	71.6589	A					
D (AIREX R82.150)	5	50.3073		B				
E (AIREX C71.75)	5	41.3822			C			
B (AIREX T92.100)	5	39.5967			C	D		
C (Balsa wood)	5	37.5435				D		
A (ROHACELL 51)	5	19.7617					E	
F (Nomex ECA_I)	5	9.4165						F

**Table 10:** Flexural modulus Tukey test results at 95% confidence.

Core Type	N	Mean	Grouping				
C (Balsa wood)	5	10,193.6	A				
G (Soric XF)	5	10,036.7	A				
D (AIREX R82.150)	5	6917.2		B			
E (AIREX C71.75)	5	5777.2			C		
B (AIREX T92.100)	5	5621.4			C		
A (ROHACELL 51)	5	4249.2				D	
F (Nomex ECA_I)	5	2596.3					E

## Data Availability

The original contributions presented in the study are included in the article; further inquiries can be directed to the corresponding authors.
